# Reproductive Strategies of the Insidious Fish Ectoparasite, *Neobenedenia* sp. (Capsalidae: Monogenea)

**DOI:** 10.1371/journal.pone.0108801

**Published:** 2014-09-29

**Authors:** Truong Dinh Hoai, Kate S. Hutson

**Affiliations:** 1 Marine Parasitology Laboratory, Centre for Sustainable Tropical Fisheries and Aquaculture and the School of Marine and Tropical Biology, James Cook University, Queensland, Australia; 2 Aquatic Environment and Fish Pathology Department, Faculty of Animal Science and Aquaculture, Vietnam National University of Agriculture, Hanoi, Vietnam; Temasek Life Sciences Laboratory, Singapore

## Abstract

Fish monogeneans are lethal parasites in aquaculture. We provide the first experimental evidence that a notorious fish monogenean, *Neobenedenia* sp., can produce viable eggs in isolation for three consecutive generations. We infected individual, isolated, farmed barramundi, *Lates calcarifer* (Bloch) with a single oncomiracidium (larva) of the hermaphroditic monogenean *Neobenedenia* sp. Isolated parasites reached sexual maturity at day 10 post-hatch (24°C, 35‰) and laid ∼3,300 embryonated eggs over 17 days. Egg production rapidly increased following sexually maturity on day 10 (58±15 eggs) and peaked on day 15 (496±68 eggs) before gradually decreasing. *Neobenedenia* sp. exhibited egg laying and egg hatching rhythms. Parasites laid eggs continuously, but egg production increased in periods of darkness (64.3%), while the majority of oncomiracidia (81%) emerged from eggs in the first three hours of light. Eggs laid by isolated ‘parent’ parasites hatched and individual emerging oncomiracidia were used to infect more individual, isolated fish, with three consecutive, isolated, parasite generations (F1, F2 and F3) raised in the laboratory. Infection success and egg hatching success did not differ between generations. Our data show that one parasite, in the absence of a mate, presents a severe threat to captive fish populations.

## Introduction

Monogeneans exhibit sophisticated life history strategies in order to ensure their survival in contrasting and unpredictable environments. Evolutionary strategies include multiple reproductive mechanisms, predator avoidance and behavioural responses to host and environmental cues that favour enhanced infection success. In wild populations these strategies ensure some parasites survive to the next generation, whereas in captive populations, where host organisms are confined in high densities, it can lead to parasite epizootics.

Various reproductive mechanisms have been observed in monogeneans including oviparity [1], viviparity [2] and self-fertilisation [3]. Most oviparous monogeneans deposit fewer than 100 eggs/parasite/day [4]–[7], although some species can produce more than 550 eggs/parasite/day [8]. In viviparous monogeneans, up to three consecutive generations can develop inside the mother parasite. For example, a single individual *Gyrodactylus salaris* bears the first daughter within 24 hours of the birth of the parent [9] and thus has the capacity to produce six million offspring in four weeks [1]. Self-fertilisation is known to occur in monogenean species that infect the bladder of amphibians [3], [10] and ensures reproductive potential when a parasite finds itself alone on a host. Monogeneans also exhibit egg laying and egg hatching rhythms, which can reduce the risk of predation and coincide with host behaviours to ensure infection success. Monogeneans maximise their chances of finding a host by releasing eggs into the environment during certain times of the day or night [7], extending the hatching period [11], responding to hatching cues such as shadows [12], chemicals [11], [13], mechanical disturbance [14]–[16] and osmotic changes [17], most of which are generated by the host.

*Neobenedenia* are marine capsalid monogeneans of critical concern to aquaculture because they exhibit several life history traits that aid their survival. *Neobenedenia* spp. have direct life cycles with short generation times [18], [19] and low host specificity [20], [21] which has resulted in major stock losses in several aquaculture fish species (see [18], [22]–[24]). Furthermore, attached parasite stages are transparent, which can reduce predation by cleaner organisms [21]. Eggs are encapsulated by a proteinaceous shell which confers protection to the developing embryo from digestion [25], most chemicals [26]–[29] and bacteria [1]. Capsalid monogeneans are hermaphrodites, displaying several types of reproduction including mutual cross-insemination [30], attachment of spermatophores to other individuals [31] and self-insemination (as observed by the copulatory organ lodged in the parasite's own uterus; see [30], [32]). However, no studies have experimentally examined whether fish monogeneans can successfully reproduce in isolation and produce viable eggs and larvae.

The aim of this research was to experimentally examine the reproductive strategies of *Neobenedenia*. Specifically we sought to determine: 1) whether *Neobenedenia* can reproduce in isolation; 2) *Neobenedenia* fecundity over time; 3) whether *Neobenedenia* exhibit egg laying rhythms and/or hatching rhythms. We used a barramundi, or Asian seabass, *Lates calcarifer* – *Neobenedenia* sp. model system for our experiments. Barramundi (Perciformes: Latidae) are distributed in estuaries and coastal seas from south-western India to north-eastern Australia, with approximate latitudes of ±25° [33]. This species is among the most important food fishes in tropical Australasia and is farmed throughout eastern and western Asia (China, India, Israel, Indonesia, Malaysia, Philippines, Singapore, Tahiti, Taiwan, Thailand) and Australia [34]. High intensities of *Neobenedenia* on farmed fish damage host epidermis through attachment and feeding [19], [23], [35] and increase secondary infections [36], [37].

## Materials and Methods

### Ethics Statement

This work was conducted using a barramundi, *Lates calcarifer* – *Neobenedenia* sp. model system with all procedures approved by the James Cook University Animal Ethics Committee (A1579). *Neobenedenia* sp. used in experiments were collected from private land in north Queensland, Australia. Future permissions should be directed to Coral Coast Barramundi Pty Ltd.

### Source of Animals

Hatchery reared freshwater *L. calcarifer*; mean size 125±23 mm) were purchased from Good Fortune Bay Hatchery, Queensland, Australia for use in experiments 1–4. Fish were not previously exposed to *Neobenedenia*. Fish were transported to the laboratory in 50 L tanks with air supplied through battery powered aerators and held in fresh water in 100 L aquaria until required. Fish were acclimated to sea water 48 h prior to experiments by increasing salinity to 5, 10, 20, 30 and 35‰ over 6 h intervals. Sea water used in experiments was UV treated, 10 µm filtered, 35‰, unless stated otherwise.

*Neobenedenia* sp. used in experiments were collected from a land-based marine *L. calcarifer* farm (Coral Coast Barramundi Pty Ltd) in north Queensland, Australia. An infection was maintained on *L. calcarifer* (size range 110–220 mm) held in 100 L marine aquaria to provide a continuous source of parasites. *Neobenedenia* sp. (hereafter as *Neobenedenia*) investigated in this study is presently unidentified given the absence of diagnostic criteria to differentiate between geographical/host isolates and species [20], [21], [38] [20], [21], [33]. Representative parasites were accessioned in the Australian Helminth Collection (AHC) at the South Australian Museum (SAMA AHC 35461; see [39]).

### Experiment 1: Reproduction of Isolated *Neobenedenia*

To determine whether hermaphroditic *Neobenedenia* can reproduce in isolation, individual, isolated fish were infected with a single oncomiracidium (larva). Oncomiracidia were sourced from embryonated *Neobenedenia* eggs collected from the laboratory infection. Eggs were incubated in glass cavity blocks in sea water at 25°C in culture chambers on a 12∶12 h LD cycle (Sanyo: ML-351 Versatile Environmental Incubation Chamber). A third of the solution (2 mL) was exchanged every 24 h and eggs were monitored daily under a stereomicroscope using both transmitted and incident light. When eye spots were observed in the eggs, monitoring was increased to every 2 h in order to obtain newly hatched oncomiracidia. Individual oncomiracidia <4 h old were gently aspirated using a fine-tip glass pipette under a stereomicroscope and introduced to a 10 L aquarium containing an individual *L. calcarifer* in 6 L of sea water. Each oncomiracidium was released at the bottom of the aquarium to avoid the effects of surface tension and currents which can trap and kill oncomiracidia [1]. When the oncomiracidia were introduced, air supply to the fish was stopped for 1 h in order to reduce water currents and thereby increase infection success [19]. Thirty replicates were made at room temperature (24.3±0.2°C). Salinity in each aquarium was checked using a refractometer every 24 h and adjusted by adding distilled water to maintain 35±1‰.

To determine the onset of egg production, a piece of 5 cm^2^ fine gauge (0.5×0.5 mm) netting was immersed in each aquarium and checked daily under a stereomicroscope. Eggs have filamentous strings which easily entangle on netting [40]. The day that eggs were observed on the netting was recorded as time to sexual maturity. Eggs always entangle on netting on the day of sexual maturity (AK Brazenor, unpublished data). Netting was renewed daily and any suspended eggs remaining in the sea water were collected daily by filtering the solution through a 60 µm mesh.

In order to confirm isolated fish were infected by a single, individual parasite, fish were immersed in fresh water at the end of the experiment, which kills *Neobenedenia* [see 35]. Fish were bathed in 1 L of fresh water containing a mild sedative (Aqui-S 1∶1000) for 5 min. The fresh water solution and the body surface of the fish were examined twice under a stereomicroscope to collect detached and attached parasites, respectively.

We sought to determine the reproductive viability of consecutive generations of reproductively isolated parasites for a single parasite lineage. Eggs laid by a randomly selected isolated ‘parent’ parasite were incubated in culture chambers (25°C, 35‰). A single oncomiracidium from these eggs was used to infect each of 15 replicate, isolated *L. calcarifer*. This process was repeated to infect 10 and 30 fish using oncomiracidia from generation F1 and F2, respectively (Table 1). Infection success was recorded as the number of oncomiracidia that attained sexual maturity from the number of fish challenged.

**Table 1:** Infection success and egg hatching success of three consecutive isolated *Neobenedenia* sp. generations infecting *Lates calcarifer*.

Source of oncomiracidia	Infection success (%)*	Parasite generation	Egg hatching success (%)
Embryonated eggs (laboratory infection)	35 (n = 40)	Parent	78 (n = 42)
From eggs laid by isolated parent parasites	44 (n = 25)	F1	75 (n = 30)
From eggs laid by isolated F1 parasites	35 (n = 20)	F2	86 (n = 12)
From eggs laid by isolated F2 parasites	56 (n = 30)	F3	Not examined

The number of replicates (n) is indicated in parentheses.

*Data combined from *Lates calcarifer* infected in Experiment 1 and 2.

### Experiment 2: Egg Hatching Success

In order to assess the viability of eggs laid by isolated parasites, egg hatching success was determined for a single parasite lineage for three consecutive, reproductively isolated, *Neobenedenia* generations. A total of 30 fish (ten for each generation) were infected as per the methods in Experiment 1. Infection success was recorded as the number of oncomiracidia that attained sexual maturity from the number of fish challenged. *Neobenedenia* eggs were collected by filtering the aquarium sea water through a 60 µm filter three days following sexual maturity. Following egg collection, individual fish were bathed in fresh water to confirm infection by an individual parasite (see Experiment 1). Clusters of eggs (containing 8–46 individual eggs) were incubated in sea water in cavity blocks in laboratory conditions (natural light, 24.3±0.1°C) with six replicate egg clusters made for each infected fish. Cavity blocks were filled with sea water to the brim and covered with a glass cover. Blocks were monitored for egg hatching every 24 h, when one third of the solution was changed with minimal disturbance to the eggs. When hatching was observed, oncomiracidia were removed with a pipette. Hatching experiments were continued until 48 h passed without hatching in any treatment. Hatching success of *Neobenedenia* eggs was measured as the number of oncomiracidia removed divided by the total number of eggs.

### Experiment 3: Fecundity of Isolated and Cross-fertile *Neobenedenia*

To determine the fecundity of isolated parasites, egg production was monitored every 24 h. Twenty-five fish were infected with an individual oncomiracidium sourced from the laboratory infection (see Experiment 1). All aquaria (10 L with 6 L sea water) were maintained in laboratory conditions (24.3±0.1°C, 35±1‰). Ten fish (40%) were successfully infected. Daily egg production was determined by filtering the aquarium sea water through a 60 µm filter at 1800 each day. Immediately prior to filtering, fish were gently removed by hand and placed into a new aquarium containing fresh sea water. Eggs were counted under a stereomicroscope using a hand held counter to determine egg production/parasite/day. The filtered sea water was also examined carefully following death and detachment of a parasite. The experiment was terminated when mean egg production was <50 eggs/day for two consecutive days. At the termination of the experiment, individual fish were bathed in fresh water to confirm infection by an individual parasite (see Experiment 1).

To determine the fecundity of parasites given an opportunity to cross-inseminate, egg production was monitored every 12 h for fish infected with multiple oncomiracidia. Groups of ten oncomiracidia were used to challenge three fish, maintained in three separate aquaria in laboratory conditions. Egg production was monitored every 12 h (at 0600 and 1800). The experiment was terminated when mean egg production was <50 eggs/day for two consecutive days. On the day the experiment was terminated, fish were bathed in fresh water and parasites were counted. Egg production in each replicate was divided by the total number of parasites recovered to determine mean number of eggs laid per parasite.

### Experiment 4: Egg Laying Rhythm

To determine whether *Neobenedenia* exhibit an egg laying rhythm, egg production was monitored every 3 h for three days. Four fish infected with an individual, isolated *Neobenedenia*, were monitored between day 12 and 15 post-infection. Ten litre aquaria containing individual infected fish were placed in culture chambers on day 10 post-infection on a 12∶12 h LD cycle at 25°C. Aquaria were aerated with battery operated aerators and salinity was maintained at 35±1‰. Egg production was determined every 3 h for three days (72 h) by filtering the aquarium sea water through a 60 µm filter, commencing at the first period following the onset of darkness (1800–2100) on day 12. Immediately prior to filtering, fish were gently removed by hand and placed into a new aquarium with fresh sea water. Eggs were counted under a stereomicroscope using a hand held counter to determine egg production/parasite/3 h. At the termination of the experiment, individual fish were bathed in fresh water to confirm infection by an individual *Neobenedenia* (see Experiment 1).

### Experiment 5: Hatching Rhythm

To determine whether *Neobenedenia* exhibit an egg hatching rhythm, hatching was observed every 3 h throughout the day and night until all eggs had hatched. Eggs were sourced from parasites infecting fish held in the laboratory infection. Individual infected fish were removed from 100 L tanks to a 10 L aquarium with fresh sea water containing pieces of 5 cm^2^ fine gauge netting for 2 h. Netting was removed and immediately placed in a Petri dish containing fresh sea water. Pieces of netting were randomly chosen and cut with dissecting scissors to obtain pieces with ∼20 to 25 entangled eggs. A piece of netting was placed in each of 10 replicate glass cavity blocks and filled with fresh sea water to the brim and sealed with a glass lid.

Cavity blocks were exposed to natural light from a window in the laboratory. Temperature (24.1±0.1°C) was measured using a Hach Temperature Meter (HQ30d/LDO101). Approximate official times of sunrise and sunset during the experiment were 0645 and 1755, respectively. Eggs were monitored daily and a third of the sea water (2 mL) was exchanged daily. When eye spots were detected on developing embryos, monitoring was increased to every 3 h. When oncomiracidia began hatching, each piece of netting was transferred to a new cavity block containing fresh sea water at 3 h intervals, throughout the day and night. During the night, netting was transferred using dim light directed away from the cavity blocks. After each transfer, a few drops of formaldehyde were added to the cavity blocks from which the netting had been removed. Formaldehyde rapidly killed any oncomiracidia that had hatched in the block in the previous 3 h period [41]. Oncomiracidia were counted under a stereomicroscope using a hand held counter. The experiment was terminated when all embryonated eggs had hatched. Non-embryonated eggs (*n* = 7) were excluded from the analyses.

Data collected for each of the five experiments described was made publically available in the James Cook University research data repository: https://research.jcu.edu.au/researchdata/default/detail/d9eda2a2d49ef0b1ab4de74a2c77c826/

### Statistical Analysis

A chi-square distribution was used to examine proportional infection success between consecutive isolated generations. Egg hatching data were analysed by permutational analysis of variance in the PERMANOVA function of PRIMER 6.0. PERMANOVA compares the observed value of a test statistic (F-ratio) against a recalculated test statistic generated from random permutation of the data. PERMANOVAs with 999 permutations based on Euclidean distance were used to test the effect of generation on hatching success with fish as a nested factor in the design.

Once egg production started in Experiment 3, fecundity increased rapidly to a peak approximately six days later, and then declined more slowly over subsequent days. This is a relatively common pattern of fecundity in invertebrate species (see [42]) and can be modelled in a variety of ways. We chose to use a model of the following form y = a x^b^e ^−cx^ (where y is the number of eggs produced, x is the reproductive age in days). The model was fitted using the nls function in SPlus/R. An ANOVA was used to determine significant difference in average fecundity between model groups. Levene's test was used to compare model fit between groups. Day 25 and 26, where mean egg production was <50 eggs/parasite/day, were excluded because less than three replicate fish remained in the isolated group. A chi-square test was used to examine the egg hatching rhythm using the proportion of eggs hatched during 3 h intervals. Significance was accepted at p<0.05.

## Results

### Multiple Generations from Isolated *Neobenedenia*

Single *Neobenedenia* infecting an individual, isolated fish, laid embryonated eggs that hatched into viable oncomiracidia. A further two consecutive, isolated, generations were reared in the laboratory (Table 1). There was no significant difference in infection success (p = 0.277) or hatching success (p = 0.723) between consecutive generations (Table 1), however, fish host had a significant effect on hatching success (p = 0.001). Following fresh water bathing, all experimental fish were confirmed to be infected with a single, individual *Neobenedenia*.

### Fecundity

**Figure 1:**
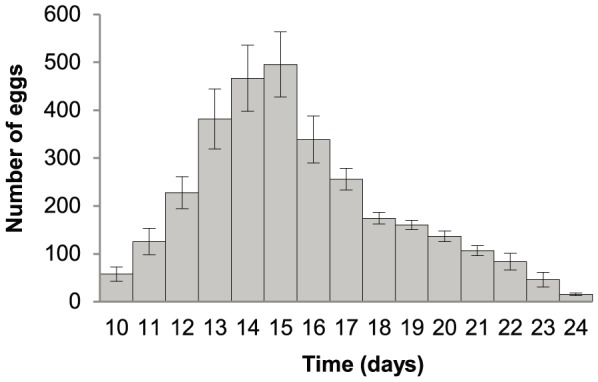
Fecundity of isolated *Neobenedenia* sp. Parasites reached sexual maturity on day 10. Error bars indicate standard error.

Individual, isolated *Neobenedenia* were fecund and produced 3,229±37 eggs/parasite over the course of the experiment (17 days of egg-laying; or 8±2 eggs/parasite/hour (e/p/h) or 190±11 eggs/parasite/day (e/p/d)). All parasites reached sexual maturity on day 10 post-infection (at 24.2±0.1°C). Egg production rapidly increased from the day of sexual maturity (58±15 on day 10) and peaked on day 15 (496±68) post-infection (Fig. 1). Following day 16, egg production gradually decreased, to almost negligible egg production on day 25 and 26. A total of eight fish mortalities occurred over the course of the experiment with 9, 8, 6, 3 and 2 fish remaining on days 12, 13, 20, 24 and 26, respectively. No dead, detached parasites were observed in filtered sea water for the duration of the experiment. Following fresh water bathing, all experimental fish (including mortalities) were confirmed to be infected with a single, individual *Neobenedenia*.

**Figure 2:**
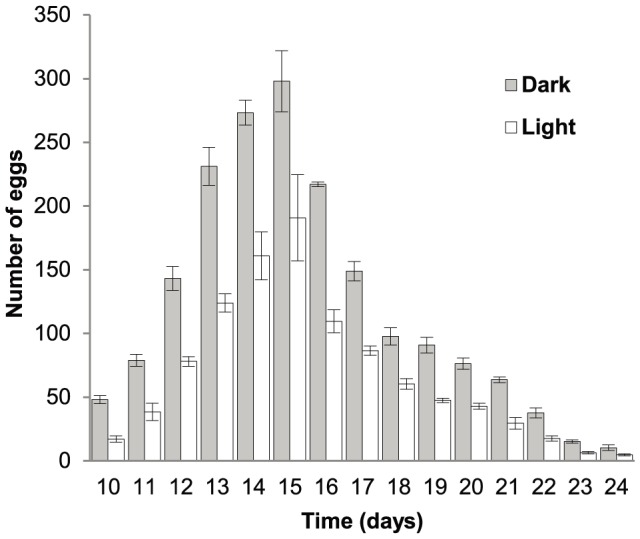
Fecundity of *Neobenedenia* sp. in 12 h periods of light and dark. Parasites reached sexual maturity on day 10 and had the opportunity to cross-fertilise. Error bars indicate standard error.

There was no significant difference between fecundity of isolated parasites and fish infected with multiple parasites (p = 0.44). Three fish challenged with multiple oncomiracidia (*n* = 0) were successfully infected with three, three and four parasites, respectively. *Neobenedenia* with the opportunity to cross-inseminate produced 8±1 e/p/h or 191±22 e/p/d or 2,865±77 eggs/parasite over the course of the experiment (15 days), with 64.3% of eggs produced during periods of darkness (Fig. 2). Parasites reached sexual maturity on day 10 post-infection (Fig. 2). Egg production rapidly increased from 66±5 on day 10 to 489±43 on day 15. On day 16, egg production began to gradually decrease, prior to almost negligible egg production on day 23 and 24 (Fig. 2). No fish mortalities occurred during the experiment, and there were no dead, detached parasites observed in filtered sea water for the duration of the experiment. Parameter estimates for isolated and cross-fertile individuals modelled separately are shown in Table 2. Although there was no significant difference in average fecundity between groups, the model better described the cross-fertile group (R^2^ = 0.93) than the isolated group (R^2^ = 0.585). Comparing the residuals of each model with a Levene's test indicated that the residual variance was significantly higher in the isolated group than the cross-fertile group (p = 0.0048).

**Table 2:** Model parameter estimates and R square values for isolated and cross-fertile *Neobenedenia* sp.

Parameter	Isolated estimate ± SE	Cross-fertile estimate ± SE
*a*	33.81±12.97	24.87±6.38
*b*	3.93±0.52	4.73±0.34
*c*	0.75±0.09	0.84±0.06
R^2^	0.585	0.93

### Egg Laying Rhythm

**Figure 3:**
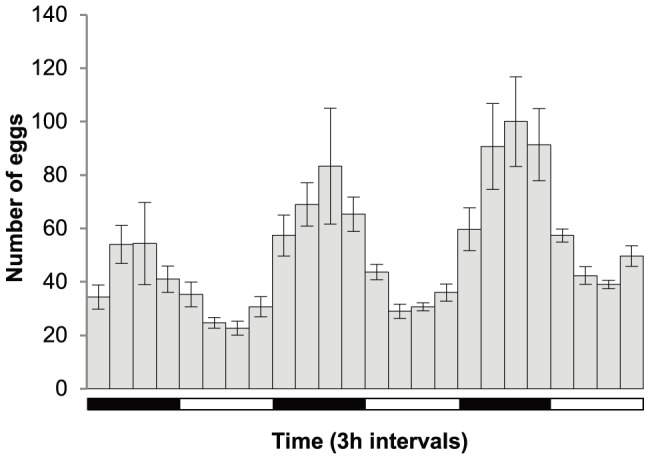
*Neobenedenia* sp. egg laying rhythm. Mean number of eggs laid by isolated *Neobenedenia* sp. (*n* = 4) shown in three hour intervals over 72 hours (from 1800 on day 12 to 1800 on day 15). Error bars indicate standard error. Horizontal panels indicate periods of light (white; 0600–1800) and dark (black; 1800–0600).

*Neobenedenia* laid eggs continuously, but exhibited a distinct egg-laying rhythm with more eggs laid during periods of darkness. Egg production began gradually increasing in the 3 h period prior to darkness and during periods of darkness, peaking between midnight and 0300 (Fig. 3). Production decreased in the 3 h period prior to illumination and the lowest egg production was between midday and 1500 (Fig. 3). Each parasite laid an average of 22±4 eggs/h in periods of darkness and 12±2 eggs/h in periods of light between day 12 and 15 post-infection.

### Egg Hatching Rhythm

**Figure 4:**
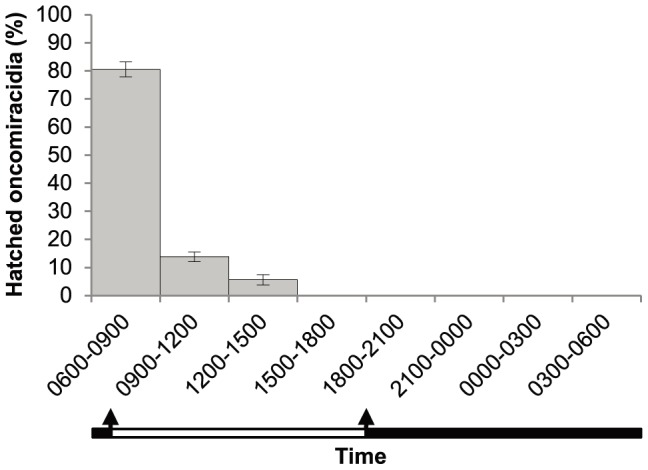
*Neobenedenia* sp. egg hatching rhythm. Percentage of eggs hatched is expressed as the sum of all oncomiracidia hatched during the same period between day 5 and 8 from the total number of oncomiracidia collected. Error bars indicate standard error. The natural period of light is indicated by the white bar and the official times of dawn and dusk (0645 and 1755) are indicated by arrows.

*Neobenedenia* exhibited a distinct hatching rhythm (p<0.001) with the majority of oncomiracidia (81%) hatching in the first 3 h of light (Fig. 4). Eggs began hatching on day 5, with the majority of hatching occurring on days 7 and 8. Egg hatching ceased on day 8. A total of 216 embryonated eggs were incubated and all eggs hatched during periods of natural light (0600–1500) with no eggs hatching between 1500 and 0600 on any day/night (Fig. 4).

## Discussion

This study provides unambiguous experimental evidence that the fish monogenean, *Neobenedenia* sp., can successfully reproduce in isolation. One isolated *Neobenedenia* has the capacity to produce more than three thousand eggs that hatch into infective larvae within two weeks (Fig. 1), revealing that low *Neobenedenia* burdens in host populations do not necessarily restrict reproductive potential. Furthermore, the progeny of isolated parasites are viable for at least two more consecutive isolated generations (Table 1). While inbreeding tends to decrease hatching and infection success and the genetic diversity of parasite populations [43], we found no significant difference in egg hatching or infection success in consecutive isolated generations (Table 1). Moreover, the nested factor of fish was significant, indicating that the relationship between parasites and their individual hosts is an important aspect in determining parasite reproductive success.

Self-fertilisation is a strategy commonly seen in parasitic platyhelminths where low parasite burdens occur in host populations or where there may be a high frequency of single parasite infection [6], [44], [45]. At least four species of monogeneans in the bladders of amphibians are capable of producing viable eggs in isolation [3], [6], [10]. It is possible that *Neobenedenia* examined in this study reproduced by natural parthenogenesis (a form of asexual production where growth of embryos can occur without fertilisation), however, this is unlikely considering that *Neobenedenia* have reproductive organs of both sexes. Kearn and Whittington [30] observed two capsalid monogenean species, *Benedeniella macrocolpa* and *B. posterocolpa*, self-inseminating in preserved specimens mounted on slides, but insemination via the vaginal route is not an option in *Neobenedenia* spp. as there is no vagina. Insemination in *Neobenedenia* spp. is most likely achieved by sperm being introduced via the common genital pore or the uterus. Indeed, Whittington and Horton [20] observed the penis of one *Neobenedenia melleni* lodged in its own uterus. Self-insemination has been observed in live specimens of *Neobenedenia girellae* (see [46]) and *Heterobothrium okamotoi* (Monogenea: Diclidophoridae) (see [32]). Furthermore, Ogawa et al. [46] suggested that self-insemination in *N. girellae* may involve passage of sperm through the tegument from externally attached spermatophores. While the specific mechanism of self-insemination was not determined, our study provides the first experimental evidence that capsalid monogeneans of fish can produce viable eggs in isolation.

Parasites that exhibit high fecundity increase the likelihood of offspring successfully locating and infecting a new host. Isolated *Neobenedenia* were fecund, with egg production rapidly increasing following sexual maturity, before peaking and slowly decreasing over time (Fig. 1). This trend is typical of many invertebrate species [47], [48]. *Neobenedenia* egg production was negligible by the time adult parasites were ∼23 days old, indicating that fecundity was captured over the reproductive life span of the parasite (Fig. 1 and 2). Egg production varied with parasite age and time of day, indicating that egg production measured on an hourly or daily rate may not accurately represent parasite fecundity ([49]; Fig. 1; Fig. 3). Egg production can also be influenced by other environmental and host variables, including temperature [50]–[52].

Parasite fecundity can vary between self-fertile and cross-fertile individuals. Wedekind *et al.* [53] reported that self-fertile cestodes, *Schistocephalus solidus*, infecting stickleback, *Gasterosteus aculeatus*, exhibited higher fecundity compared to parasites placed in pairs. In contrast, Tinsley and Owen [3] found that multiple infections of the monogenean *Protopolystoma xenopodis* infecting the toad, *Bufo regularis*, sometimes resulted in greater output per individual than isolated parasites. In our study, there was no significant difference in fecundity for isolated and cross-fertile *Neobenedenia*. It is plausible that *Neobenedenia* may not have cross-fertilised, despite being infected with multiple individuals and their ability to ‘crawl’ along the external surfaces of fishes in order to locate another parasite [54]. Thus, molecular methods are warranted to quantify the frequency of cross-fertilisation in *Neobenedenia*.

Parasite egg laying rhythms could be a predator avoidance behaviour and could also align with temporal host behaviours [25]. *Neobenedenia* laid eggs continuously, but significantly more eggs (64.3%) were laid at night (Fig. 3 & 4). Egg-laying rhythms are common in many invertebrates, with most releasing their gametes during periods of darkness (Fig. 3; [55], [56], [57]). The egg laying rhythm of *Diplozoon homoion gracile* (Monogenea: Diplozoidae), a gill parasite of southern barbel, *Barbus meridionalis*, is also nocturnal [7]. Similarly, Mooney et al. [51] found that *Heteraxine heterocerca* (Monogenea: Heteraxinidae) a gill parasite of Japanese yellowtail, *Seriola quinqueradiata*, laid eggs continuously, but more eggs (72.9%) were laid during periods of darkness, with the majority of eggs released during the first 3 h periods immediately after dark. Alternatively, some monogenean species store their eggs *in utero* to be released at a specific time of day [8], [51].

Hatching rhythms can also increase the chances of larvae contacting a specific host [58], [54]. This is crucial for infection success because free swimming oncomiracidia are typically short lived (24 to 48 h) [1], [26], [59]. *Neobenedenia* exhibited a distinct hatching rhythm, with 81% of the larvae emerging during the first 3 h of natural light (Fig. 4). Monogeneans can maximise their chances of finding a host by extending the hatching period [11], while some species respond to hatching cues such as shadows [12], chemicals [11], [13], mechanical disturbance [14]–[16] and osmotic changes [17], most of which are generated by the host. Hatching rhythms have been documented in other marine monogeneans in the first few hours of light (*Entobdella solea* [60]; *Diclidophora* spp. [15]) and also in the first few hours following dusk (*Entobdella hippoglossi* [61]; *Diplozoon homoion gracile* [7]). Other species exhibit more complicated rhythms [41], [58], [62], [63]. Hatching rhythms of monogeneans are often related to times when the behaviour of the host makes it more vulnerable to infection [7], [15], [59]–[62]. Eggs also hatch in response to other environmental cues such as light periodicity and intensity [60], [64], host skin secretions [65], mechanical disturbances [66] and seasonal differences [15]. In addition, rhythmic hatching may minimise predation on monogeneans by other organisms, especially filter feeders [58], [63].

In conclusion, *Neobenedenia* exhibits a variety of strategies to aid survival of subsequent generations. Parasites can reproduce in isolation to produce viable, infective oncomiracidia for three consecutive generations. High fecundity, egg laying and egg hatching rhythms ensure the success and persistence of this harmful parasite in wild and farmed fishes.

## References

[1] Whittington ID, Chisholm LA (2008) Diseases caused by Monogenea. In: Eiras J C, Segner H, Wahlii T & Kapoor BG Editors. Fish Diseases. Science Publishers, Inc, New Hampshire, USA. pp. 683–816.

[2] Harris P, Tinsley R (1987) The biology of *Gyrdicotylus gallieni* (Gyrodactylidea), an unusual viviparous monogenean from the African clawed toad, *Xenopus laevis* . Journal of Zoology 212: 325–346.

[3] Tinsley R, Owen RW (1975) Studies on the biology of *Protopolystoma xenopodis* (Monogenoidea): the oncomiracidium and life-cycle. Parasitology 71: 445–463.

[4] Tinsley R (1983) Ovoviviparity in platyhelminth life-cycles. Parasitology 86: 161–196. 6346232 10.1017/s0031182000050885

[5] Kearn G (1985) Observations on egg production in the monogenean (*Entobdella soleae*). International Journal for Parasitology 15: 187–194.

[6] Jackson HC, Tinsley R (1988) The capacity for viable egg production by the monogenean *Protopolystoma xenopodis* in single and multiple infections. International Journal for Parasitology 18: 585–589.

[7] Macdonald S, Jones A (1978) Egg-laying and hatching rhythms in the monogenean *Diplozoon homoion gracile* from the southern barbel (*Barbus meridionalis*). Journal of Helminthology 52: 23–28. 659823 10.1017/s0022149x00005071

[8] Mooney AJ, Ernst I, Whittington ID (2006) An egg-laying rhythm in *Zeuxapta seriolae* (Monogenea: Heteraxinidae), a gill parasite of yellowtail kingfish (*Seriola lalandi*). Aquaculture 253: 10–16.

[9] Scott ME (1982) Reproductive potential of *Gyrodactylus bullatarudis* (Monogenea) on guppies (*Poecilia reticulata*). Parasitology 85: 217–236.

[10] Combes C (1972) Influence of the behaviour of amphibians on helminth life cycles. Zool J Linn Soc 51: 151–170.

[11] Macdonald S (1974) Host skin mucus as a hatching stimulant in *Acanthocotyle lobianchi*, a monogenean from the skin of *Raja* spp. Parasitology 68: 331–338. 4472138 10.1017/s0031182000045868

[12] Gannicott A, Tinsley R (1997) Egg hatching in the monogenean gill parasite *Discocotyle sagittata* from the rainbow trout (*Oncorhynchus mykiss*). Parasitology 114: 569–579.

[13] Whittington I (1987) Hatching in two monogenean parasites from the common dogfish (*Scyliorhinus canicula*): the polyopisthocotylean gill parasite, *Hexabothrium appendiculatum* and the microbothriid skin parasite, *Leptocotyle minor* . Journal of the Marine Biological Association of the United Kingdom 67: 729–756.

[14] Glennon V, Chisholm LA, Whittington ID (2006) Three unrelated species, 3 sites, same host–monogenean parasites of the southern fiddler ray, *Trygonorrhina fasciata*, in South Australia: egg hatching strategies and larval behaviour. Parasitology 133: 55–66. 16563201 10.1017/S003118200600998X

[15] Macdonald S (1975) Hatching rhythms in three species of *Diclidophora* (Monogenea) with observations on host behaviour. Parasitology 71: 211–228. 1187182 10.1017/s0031182000046667

[16] Whittington ID, Cribb BW, Hamwood TE, Halliday JA (2000) Host-specificity of monogenean (platyhelminth) parasites: a role for anterior adhesive areas? International Journal for Parasitology 30: 305–320. 10719124 10.1016/s0020-7519(00)00006-0

[17] Tinsley R (1978) Oviposition, hatching and the oncomiracidium of *Eupolystoma anterorchis* (Monogenoidea). Parasitology 77: 121–132.

[18] Hirazawa N, Mitsuboshi T, Hirata T, Shirasu K (2004) Susceptibility of spotted halibut *Verasper variegatus* (Pleuronectidae) to infection by the monogenean *Neobenedenia girellae* (Capsalidae) and oral therapy trials using praziquantel. Aquaculture 238: 83–95.

[19] Hirazawa N, Takano R, Hagiwara H, Noguchi M, Narita M (2010) The influence of different water temperatures on *Neobenedenia girellae* (Monogenea) infection, parasite growth, egg production and emerging second generation on amberjack *Seriola dumerili* (Carangidae) and the histopathological effect of this parasite on fish skin. Aquaculture 299: 2–7.

[20] Whittington I, Horton M (1996) A revision of *Neobenedenia Yamaguti*, 1963 (Monogenea: Capsalidae) including a redescription of *N. melleni* (MacCallum, 1927) Yamaguti, 1963. Journal of Natural History 30: 1113–1156.

[21] Whittington ID (2012) *Benedenia seriolae* and *Neobenedenia* species. In: Woo, PTK and Buchmann, K editors. Fish Parasites: Pathobiology and Protection. CABI, Wallingford, Oxfordshire, UK. pp. 225–244.

[22] Deveney MR, Chisholm LA, Whittington ID (2001) First published record of the pathogenic monogenean parasite *Neobenedenia melleni* (Capsalidae) from Australia. Diseases of Aquatic Organisms 46: 79–82. 11592706 10.3354/dao046079

[23] Ogawa K, Miyamoto J, Wang HC, Lo CF, Kou GH (2006) *Neobenedenia girellae* (Monogenea) infection of cultured cobia *Rachycentron canadum* in Taiwan. Fish Pathology 41: 51–56.

[24] Ogawa K, Yokoyama H (1998) Parasitic diseases of cultured marine fish in Japan. Fish Pathology (Japan) 33: 303–309.

[25] Kearn GC (1986) The eggs of monogeneans. Advances in Parasitology 25: 175–273. 3535435 10.1016/s0065-308x(08)60344-9

[26] Militz TA, Southgate PC, Carton AG, Hutson KS (2014) Efficacy of garlic (*Allium sativum*) extract applied as a therapeutic immersion treatment for *Neobenedenia* sp. management in aquaculture. Journal of Fish Diseases 37: 451–461. 23952605 10.1111/jfd.12129

[27] Thoney D (1990) The effects of trichlorfon, praziquantel and copper sulphate on various stages of the monogenean *Benedeniella posterocolpa*, a skin parasite of the cownose ray, *Rhinoptera bonasus* (Mitchill). Journal of Fish Diseases 13: 385–389.

[28] Wharton D (1983) The production and functional morphology of helminth egg-shells. Parasitology 86: 85–97. 6346235 10.1017/s003118200005085x

[29] Yoshinaga T, Segawa I, Kamaishi T, Sorimachi M (2000) Effects of temperature, salinity and chlorine treatment on egg hatching of the monogenean *Neoheterobothrium hirame* infecting Japanese flounder. Fish Pathology 35: 85–88.

[30] Kearn G, Whittington I (1992) Diversity of reproductive behaviour in platyhelminth parasites: insemination in some benedeniine (capsalid) monogeneans. Parasitology 104: 489–496.

[31] Kearn G (1970) The production, transfer and assimilation of spermatophores by *Entobdella soleae*, a monogenean skin parasite of the common sole. Parasitology 60: 301–311. 5464024 10.1017/s0031182000078136

[32] Ogawa K (2002) Impacts of diclidophorid monogenean infections on fisheries in Japan. International Journal for Parasitology 32: 373–380. 11835977 10.1016/s0020-7519(01)00338-1

[33] Pethiyagoda R, Gill AC (2013) Taxonomy and Distribution of Indo-Pacific Lates. Biology and Culture of Asian Seabass *Lates calcarifer* In: Jerry DR Editor. Biology and Culture of Asian Seabass *Lates calcarifer*. CRC Press, Taylor and Francis Group, USA. pp 1–15.

[34] Hutson KS (2013) Infectious diseases of Asian seabass and health management. In: Jerry DR Editor. Biology and Culture of Asian Seabass *Lates calcarifer*. CRC Press, Taylor and Francis Group, USA. pp 102–136.

[35] Kaneko JJ, Yamada R, Brock J, Nakamura R (1988) Infection of tilapia, *Oreochromis mossambicus* (Trewavas), by a marine monogenean, *Neobenedenia melleni* (MacCallum, 1927) Yamaguti, 1963 in Kaneohe Bay, Hawaii, USA, and its treatment. Journal of Fish Diseases 11: 295–300.

[36] Thoney D, Hargis W Jr (1991) Monogenea (Platyhelminthes) as hazards for fish in confinement. Annual Review of Fish Diseases 1: 133–153.

[37] Leong T, Colorni A (2002) Infection diseases of warmwater fish in marine and brackish waters. Diseases and Disorders of Finfish in Cage Culture London 193–230.

[38] Whittington ID (2004) The Capsalidae (Monogenea: Monopisthocotylea): a review of diversity, classification and phylogeny with a note about species complexes. Folia Parasitologica 51: 109–122. 15357390 10.14411/fp.2004.016

[39] Hutson KS, Mata L, Paul NA, de Nys R (2012) Seaweed extracts as a natural control against the monogenean ectoparasite, *Neobenedenia* sp., infecting farmed barramundi (*Lates calcarifer*). International Journal for Parasitology 42: 1135–1141. 23068914 10.1016/j.ijpara.2012.09.007

[40] Kearn G, Ogawa K, Maeno Y (1992) Egg production, the oncomiracidium and larval development of *Benedenia seriolae*, a skin parasite of the yellowtail, *Seriola quinqueradiata*, in Japan. Publications of the Seto Marine Biological Laboratory 35: 351–362.

[41] Kearn GC, Ogawa K, Maeno Y (1992) Hatching Patterns of the Monogenean Parasites *Benedenia seriolae* and *Heteraxine heterocerca* from the Skin and Gills, Respectively, of the Same Host Fish, *Seriola quinqueradiata* (Communication)(Developmental Biology). Zoological Science 9: 451–455.

[42] Cushman JH, Boggs CL, Weiss SB, Murphy DD, Harvey AW, et al (1994) Estimating female reproductive success of a threatened butterfly: influence of emrgence time and hostplant phenology. Oecologia 99: 194–200. 28313966 10.1007/BF00317101

[43] Poulin R (2007) Are there general laws in parasite ecology? Parasitology 134: 763–776. 17234043 10.1017/S0031182006002150

[44] Haag KL, Araujo AMd, Gottstein B, Siles-Lucas M, Thompson R, et al (1999) Breeding systems in *Echinococcus granulosus* (Cestoda; Taeniidae): selfing or outcrossing? Parasitology 118: 63–71. 10070663 10.1017/s0031182098003485

[45] Stunkard HW (1957) Intraspecific variation in parasitic flatworms. Systematic Zoology 6: 7–18.

[46] Ogawa K, Shirakashi S, Ishitani H (2014) Insemination of the monogenean *Neobenedenia girellae* (Capsalidae, Benedeniinae). Parasitology international 63: 473–478. 24513796 10.1016/j.parint.2013.10.009

[47] Novoseltsev VN, Novoseltseva JA, Boyko SI, Yashin AI (2003) What fecundity patterns indicate about aging and longevity: insights from Drosophila studies. The Journals of Gerontology Series A: Biological Sciences and Medical Sciences 58: B484–B494. 10.1093/gerona/58.6.b484 12807919

[48] Snell TW, King CE (1977) Lifespan and fecundity patterns in rotifers: the cost of reproduction. Evolution 882–890. 28563718 10.1111/j.1558-5646.1977.tb01082.x

[49] Whittington ID (1997) Reproduction and host-location among the parasitic Platyhelminthes. International Journal for Parasitology 27: 705–714. 9229253 10.1016/s0020-7519(97)00012-x

[50] Kearn G, Ogawa K, Maeno Y (1992) The oncomiracidium of *Heteraxine heterocerca*, a monogenean gill parasite of the yellowtail *Seriola quinqueradiata* . Publications of the Seto Marine Biological Laboratory 35: 347–350.

[51] Mooney AJ, Ernst I, Whittington ID (2008) Egg-laying patterns and in vivo egg production in the monogenean parasites *Heteraxine heterocerca* and *Benedenia seriolae* from Japanese yellowtail *Seriola quinqueradiata* . Parasitology 135: 1295–1302. 18700993 10.1017/S0031182008004691

[52] Tubbs L, Poortenaar C, Sewell M, Diggles B (2005) Effects of temperature on fecundity in vitro, egg hatching and reproductive development of *Benedenia seriolae* and *Zeuxapta seriolae* (Monogenea) parasitic on yellowtail kingfish *Seriola lalandi* . International Journal for Parasitology 35: 315–327. 15722083 10.1016/j.ijpara.2004.11.008

[53] Wedekind C, Strahm D, Schärer L (1998) Evidence for strategic egg production in a hermaphroditic cestode. Parasitology 117: 373–382. 9820859 10.1017/s0031182098003114

[54] Whittington ID, Ernst I (2002) Migration, site-specificity and development of *Benedenia lutjani*(Monogenea: Capsalidae) on the surface of its host, *Lutjanus carponotatus* (Pisces: Lutjanidae). Parasitology 124: 423–434. 12003066 10.1017/s0031182001001287

[55] Ganias K, Somarakis S, Machias A, Theodorou A (2003) Evaluation of spawning frequency in a *Mediterranean sardine* population (*Sardina pilchardus sardina*). Marine Biology 142: 1169–1179.

[56] Oliver J, Babcock R (1992) Aspects of the fertilization ecology of broadcast spawning corals: sperm dilution effects and in situ measurements of fertilization. The Biological Bulletin 183: 409–417. 29300507 10.2307/1542017

[57] Taylor MH, Leach GJ, DiMichele L, Levitan WM, Jacob WF (1979) Lunar spawning cycle in the mummichog, *Fundulus heteroclitus* (Pisces: Cyprinodontidae). Copeia 1979: 291–297.

[58] Ernst I, Whittington ID (1996) Hatching rhythms in the capsalid monogeneans *Benedenia lutjani* from the skin and *B. rohdei* from the gills of *Lutjanus carponotatus* at Heron Island, Queensland, Australia. International Journal for Parasitology 26: 1191–1204. 9024862 10.1016/s0020-7519(96)00118-x

[59] Whittington ID, Kearn GC (2011) Hatching strategies in monogenean (platyhelminth) parasites that facilitate host infection. Integrative and Comparative Biology 51: 91–99. 21558179 10.1093/icb/icr003

[60] Kearn G (1973) An endogenous circadian hatching rhythm in the monogenean skin parasite *Entobdella soleae*, and its relationship to the activity rhythm of the host (*Solea solea*). Parasitology 66: 101–122. 4799083 10.1017/s0031182000044486

[61] Kearn GC (1974) Nocturnal hatching in the monogenean skin parasite *Entobdella hippoglossi* from the halibut, *Hippoglossus hippoglossus* . Parasitology 68: 161–172. 4857039

[62] Macdonald S, Combes C (1978) The hatching rhythm of *Polystoma integerrimum*, a monogenean from the frog *Rana temporaria* . Chronobiologia 5: 277. 309814

[63] Whittington I, Kearn G (1986) Rhythmical hatching and oncomiracidial behaviour in the hexabothriid monogenean *Rajonchocotyle emarginata* from the gills of *Raja* spp. Journal of the Marine Biological Association of the United Kingdom 66: 93–111.

[64] Whittington ID, Kearn GC (1989) Rapid hatching induced by light intensity reduction in the polyopisthocotylean monogenean *Plectanocotyle gurnardi* from the gills of gurnards (Triglidae), with observations on the anatomy and behaviour of the oncomiracidium. Journal of the Marine Biological Association of the United Kingdom 69: 609–624.

[65] Kearn GC (1974) The effects of fish skin mucus on hatching in the monogenean parasite *Entobdella soleae* from the skin of the common sole, *Solea solea* . Parasitology 68: 173–188. 4857040

[66] Whittington ID, Kearn GC (1988) Rapid hatching of mechanically-disturbed eggs of the monogenean gill parasite *Diclidophora luscae*, with observations on sedimentation of egg bundles. International Journal for Parasitology 18: 847–852.

