# Correction: Reproductive Strategies of the Insidious Fish Ectoparasite, *Neobenedenia* sp. (Capsalidae: Monogenea)

**DOI:** 10.1371/journal.pone.0117881

**Published:** 2015-02-10

**Authors:** The PLOS ONE Staff

There is an error in the third and fourth sentences of the Abstract. There should be a full stop after *Neobenedenia* sp.. The correct sentences are: We infected individual, isolated, farmed barramundi, *Lates calcarifer* (Bloch) with a single oncomiracidium (larva) of the hermaphroditic monogenean *Neobenedenia* sp.. Isolated parasites reached sexual maturity at day 10 post-hatch (24°C, 35‰) and laid ~3,300 embryonated eggs over 17 days.

There is an error in the second sentence of the second paragraph in the Fecundity section of the Results. The n value is incorrect. The correct sentence is: Three fish challenged with multiple oncomiracidia (n = 10) were successfully infected with three, three and four parasites, respectively.

Both errors occurred when the manuscript was being typeset for publication. The publisher apologizes for these errors.
